# Optimal Design and Testing of a Thermoplastic Pressurized Passenger Door Manufactured Using Thermoforming

**DOI:** 10.3390/polym13193394

**Published:** 2021-10-02

**Authors:** Roman Růžek, Josef Krena, Radek Doubrava, Josef Tkadlec, Martin Kadlec, Petr Bělský

**Affiliations:** 1 Materials and Technologies Department, VZLU-Czech Aerospace Research Centre, 199 05 Prague, 130 Beranových, Czech Republic; doubrava@vzlu.cz (R.D.); kadlec@vzlu.cz (M.K.); belsky@vzlu.cz (P.B.); 2 Latécoère Czech Republic, 199 02 Praha 9, 65 Beranových, Czech Republic; josef.krena@latecoere.aero (J.K.); josef.tkadlec@latecoere.aero (J.T.)

**Keywords:** composites, thermoplastics, door, damage tolerance, static, fatigue, non-destructive testing, numerical analysis

## Abstract

The present paper documents and discusses research work associated with a newly designed passenger door structure demonstrator. The composite structure was manufactured from carbon-fiber-reinforced thermoplastic resin. A composite frame with a variable cross-section was designed, optimized, and fabricated using thermoforming technology. Both numerical simulations and experiments supported structural verification according to the damage tolerance philosophy; i.e., impact damage is presented. The Tsai-Wu and maximal stress criteria were used for damage analysis of the composite parts. Topological optimization of the metal hinges from the point of view of weight reduction was used. All expected parameters and proposed requirements of the mechanical properties were proved and completed. The door panel showed an expected numerically evaluated residual strength (ultimate structure load) as well as meeting airworthiness requirements. No impact damage propagation in the composite parts was observed during mechanical tests, even though visible impact damage was introduced into the structure. No significant difference between the numerical simulations and the experimentally measured total deformation was observed. Repeated deformation measurements during fatigue showed a nonlinear structure behavior. This can be attributed to the relaxation of thermoplastics.

## 1. Introduction

At present, aeronautical industry composite structures are still primarily thermosets; nevertheless, the share of high-performance thermoplastic composites (TPCs) is continuously increasing despite their high purchase prices [1]. They provide good material properties, such as fracture and impact resistance [2,3], formability [4,5], weldability [6,7], self-healing possibilities [8,9], and recyclability [10]. The main benefits of TPCs can be seen in their potential for repeated heating and molding, without negative influence on mechanical and physical properties [11]. The curing process is completely reversible. 

Traditionally, composite fibers within a layer have the same orientation, leading to constant stiffness properties. Recently, due to the development of advanced manufacturing technologies, such as automated laying processes (fiber placement, fiber patch placement, tow shearing), the fiber orientation of a layer can be continuously varied, together with varying stiffness properties [12]. Another procedure is to align fibers in the direction of the principal stress with the aim of reducing stress concentrations and weight [13]. In [14], a three-step approach for variable-stiffness laminate design was applied to shear panels. The first step is to find the optimal stiffness distribution in terms of the lamination parameters [15,16]; the second step is to find the optimal manufacturable fiber angle distribution [17,18]; and the third step is to retrieve the fiber paths, as discussed by Blom [19]. All abovementioned methods have relatively significant disadvantages in terms of time and a costly manufacturing process. Additionally, utilization of these methods is limited by the requirements to exclude the fiber/tow shift and gaps or overlaps that are present during manufacturing [20,21].

Thermoplastics, together with thermoforming, create possibilities to manufacture flat plates with predefined fiber/fabric layer orientations, and/or tailored blanks to achieve structural parts with varying stiffnesses and mechanical properties [22,23]. The thermoforming process enables the manufacture of a structural part with varying cross sections in a relatively very short amount of time. Thus, the thermoforming process appears to be a very promising manufacturing technology for TPCs. An additional significant impact of thermoplastic material exploitation is out-of-autoclave manufacturing process development (which can potentially save up to 75% on energy consumption compared with the autoclave process) as well as weight and waste reductions [24]. All these attributes lead to a significant reduction in eco-impact throughout the life cycle of a structure. These are the main reasons why this technology and type of material were used for the pressurised passenger door that was designed, optimized, manufactured, and verified in this work. Moreover, the above-discussed advanced technology is in accordance with composite roadmap developments within the Latécoère Innovation department.

In compliance with airworthiness requirements, primary structures manufactured from composite materials must be designed and operated in compliance with the damage tolerance (DT) philosophy [25,26,27,28,29]. This approach allows, in certain cases, structure operations with an allowable size of flaw (damage). The experimental certification procedure of a structure, according to DT philosophy, requires conducting several relatively independent phases: mechanical loading (both static and fatigue), environmental loading, non-destructive inspection, simulation of impact damage, and residual static tests [30]. Environmental effects could be included as a so-called knock-down factor, which includes not only the influence of humidity on structure properties, but also structure performance at different altitudes [31]. The present paper documents and discusses relevant procedures associated with a pressurised passenger door demonstrator, designed and manufactured from carbon-fiber-reinforced composite with thermoplastic resin.

The work consisted of several phases: technology development, process optimization, numerical model development and verification, test campaign definition, loading system design and manufacturing, impact damage tests, mechanical loading, non-destructive inspection, deformation and strain measurements, and residual static strength evaluation. 

A new numerically verified manufacturing technology was developed where a composite frame with variable cross section was fabricated using thermoforming technology. The design was supported by an experimental campaign with the aim of verifying mechanical properties and comparing them to numerical simulations. The verification methodology was designed based on the damage tolerance philosophy; this means that structural properties were proofed under both static and fatigue loading conditions with the presence of impact damage. 

Numerical simulations of finite element (FE) models were used to determine the load of the real structure, to optimize the design of critical parts, and to define the representative load distribution into the newly designed door panel. A linear analysis with linear contact between each part of the structure or the load system was used for numerical simulations. The Tsai–Wu and maximal stress criteria were used for damage analysis of the composite parts. Topological optimization was used for the design of metal hinges from the point of view of weight reduction. A FE model of the real test assembly was also used for post-test analyses and results comparisons. The simplification of overpressure simulation for experimental verification of the door structure was designed and applied.

## 2. Materials

The panel consisted of the skin, three Omega beams, two Z frames, and six stop fittings (locks) assembly. Individual parts of the structure were joined using bolts. 

The Z-frame and Omega profiles were fabricated using a hot forming method. All composite parts were fabricated from carbon material utilizing a polyphenylenesulfide (PPS) thermoplastic resin system which was supplied by Toray and specified as TC1100. The melting temperature of PPS is 280 °C and that of *T*_g_ is 90 °C. The reinforcement fabric was 5HS 3K T300 with an area weight of 285 g/m^2^ and the rate of fiber volume was 50%. The Omega profiles had 10 plies, C fabric without a glass layer, and layup of [[(0,90)/(±45)]2/(0,90)]s). Z-frames had a layup of [[(0, 90)/(±45)]3/(0/90)]s) with a thin glass layer on the surface. The skin was cut using water jet technology from a flat plate with a layup of [(0,90)/(±45)]5/(0,90)). An overview of the used thermoplastic material is summarized in Table 1.

Stop fittings were milled from titanium alloy (Ti-6Al-4V) annealed plate material (minimum ultimate strength of 900 MPa, yield strength of 830 MPa). Individual parts of the door panel were joined using bolts. The Omega profiles with the stop fittings were joined using NAS6604D16 steel bolts, the Omega profiles and Z-profiles were joined using ABS0114-4 titanium bolts, and the skin, Omega profiles, and Z-profiles were joined using EN6114V3-5 titanium bolts.

**Figure 1:**
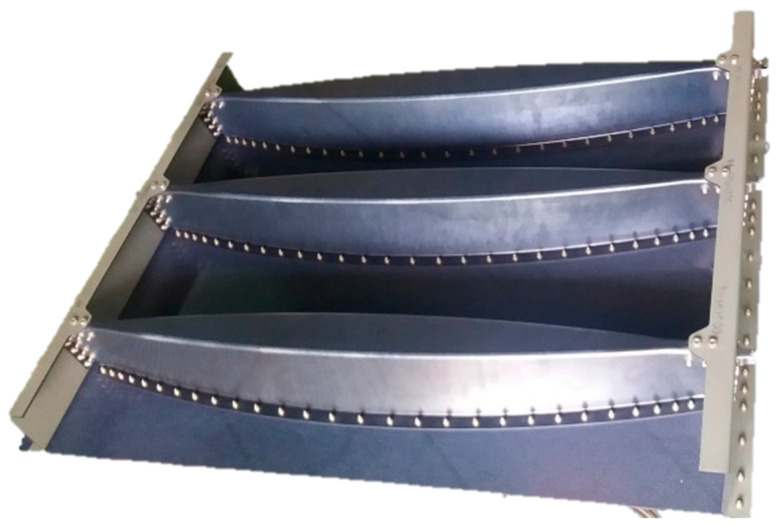
Door panel with Omega beams and Z frame.

The overall geometry of the door panel was: length (L) = 1250 mm, width (W) = 950 mm and height (H) = 114 mm. The panel was manufactured as shown in Figure 1.

## 3. Structure Optimization

### 3.1. Manufacturing Technology

**Figure 2:**
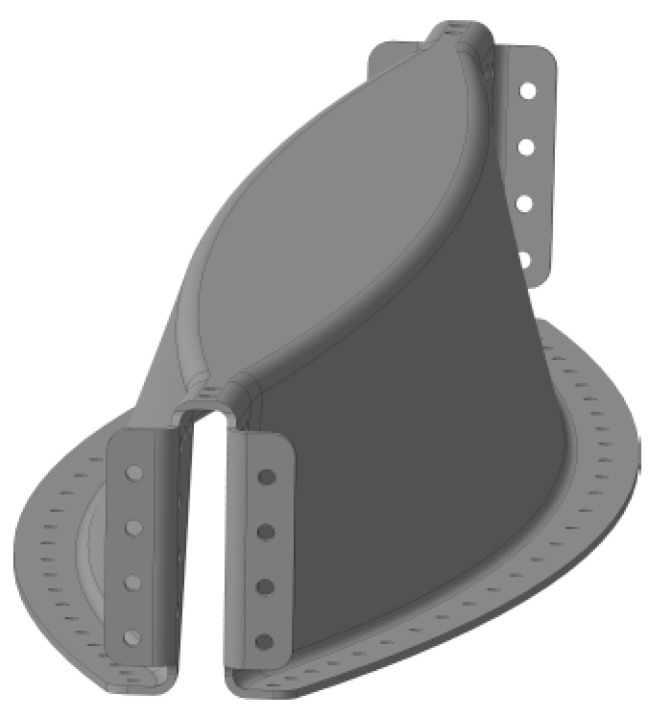
Optimized Omega profile.

The assembly consisted of three types of parts, where the Omega profile had the most complex shape. That shape was chosen due to the loading along that profile. There was maximal bending in the center of the profile, so that the maximal inertia of the cross section was located there, and the profile was wide. The opposite situation occurred on the edges of profile where the maximal shear force was located at the metal stop fitting. Here, we needed to distribute the large local force into the surrounding structure. This was why the Omega profile was designed with such a complex shape (Figure 2). 

**Figure 3:**
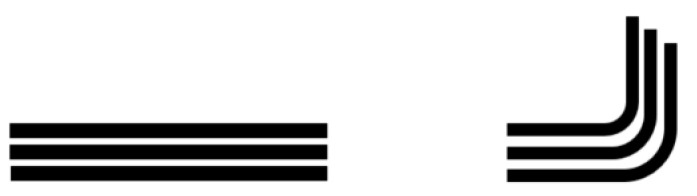
Interlaminar sliding caused by laminate folding.

**Figure 4:**
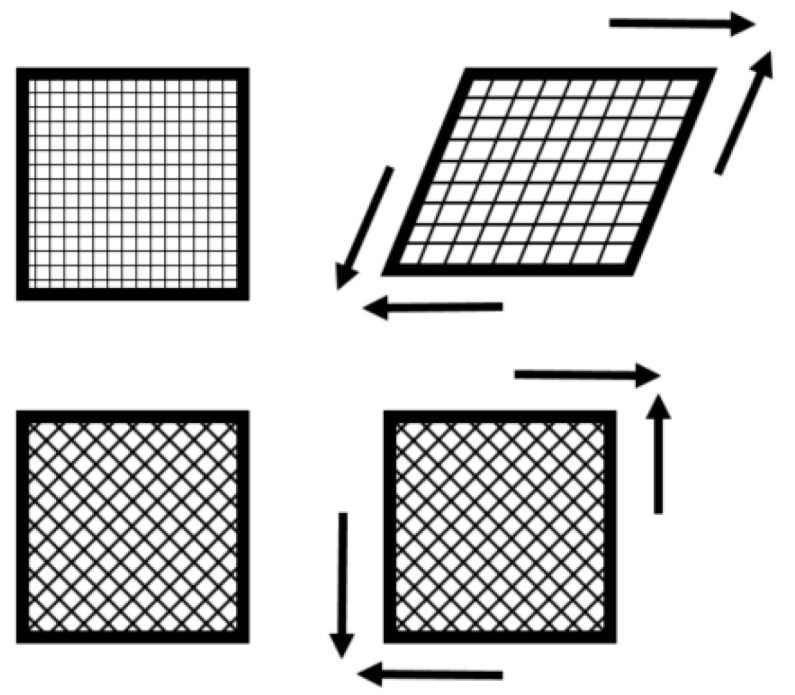
Shear deformation of the layers with different fiber orientations.

The problem of non-developable shape thermoforming is a risk to the folded layers or the wrinkles created during forming. Two basic mechanisms can help to successfully form the complex shape of a laminate. The first is sliding between layers and the second is shear deformation of the layer. Interlaminar sliding can be caused by folding of the laminate into the final shape of the part (Figure 3). Planar shear deformation also enables fitting of the laminate to a final shape; however, shear stiffness strongly depends on the orientation of fibers relative to the load direction. It also induces sliding between layers with different fiber orientations (Figure 4). This second mechanism is the key for forming non-developable parts.

**Figure 5:**
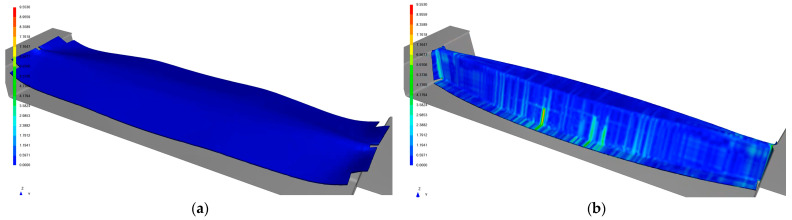
Contour maps of shear deformation at the beginning of forming (**a**) and in the final shape of stamping (**b**).

All mechanisms included special SW AniForm [32], which was used for simulations of the thermoforming process. It enables prediction, not only of wrinkles, but also reorientation of fibers, stress in layers, etc. The complex shape of the Omega beam was created on the basis of loading, as described above. However, slight modification using simulations was needed for it to be feasible to produce the beams without wrinkles and folding due to the non-developable shape. Two extracted steps in the forming simulation are displayed in Figure 5. The first step (a) shows us the shape of the blank at the beginning of forming, and the second step (b) is a fully shaped part with a spectrum displaying shear deformations.

The verified shape of the part was the baseline for designing the stamping tool, which consisted of male and female metal parts. Stamping was performed after preheating of the blank up to a process temperature of about 330 °C. The manufacture of the part confirmed the correct prediction as a good quality of profiles was achieved. 

Trimming of the contour was performed using a NC machine and assembly was carried out with the help of titanium mechanical fasteners (Hi-Lock), excluding the joining of titanium stop fittings where titanium screws were used. 

### 3.2. Load System Design

**Figure 6:**
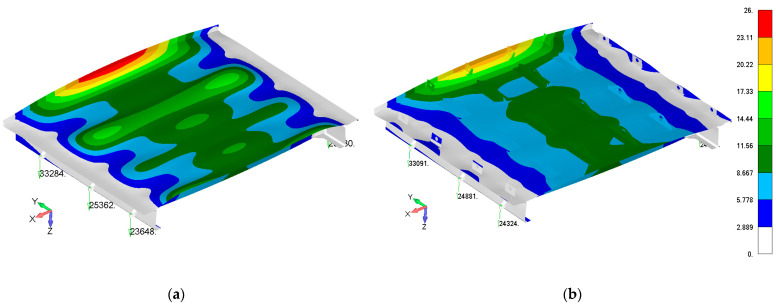
Comparison of the reference overpressure deformation map in mm (**a**) with a substitute points load (**b**) of the door panel.

**Figure 7:**
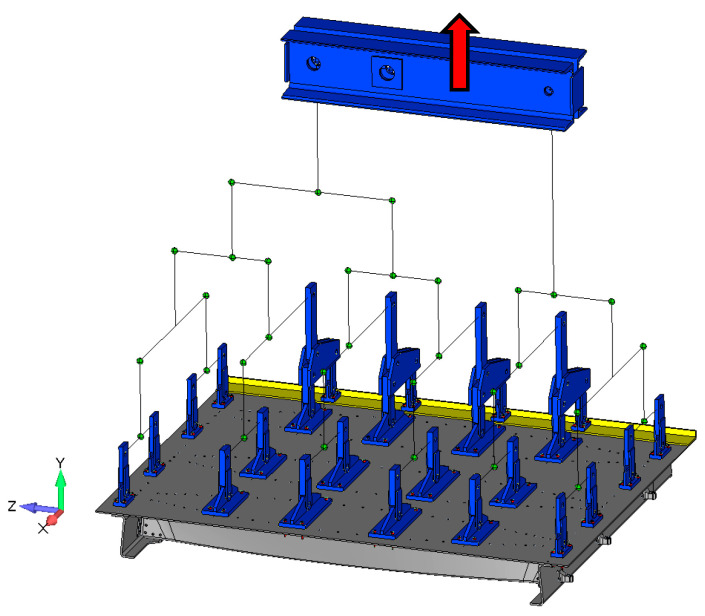
CAD model of the whiffle tree system for load distribution into the door panel.

The main load acting on the door structure was overpressure. For simulations of real pressure loads on the physical test, several variants, such as rubber bags, foam boards, special inserts, and using existing fasteners points, were considered. Finally, the continuous pressure load was transformed into the resultant force and the continuous load was introduced into the door structure using a special whiffle tree loading system. The overpressure load was distributed (pressure simulation) into the door panel through 24 individual points. The number, location, and load transfer of the individual points were optimized regarding how to achieve the best fit with a real pressure distribution. Optimization criteria were total deformation, reaction forces in stop fittings, maximum relative deformation (limit 4500 strain), and failure criterion, stress maximum, and Tsai–Wu criterion [33]. Figure 6 compares the real reference overpressure deformation map with a deformation map of the optimized loading through individual points. A scheme of the whiffle tree system is shown in Figure 7. 

## 4. Methods

The experimental procedures complied with airworthiness regulations. The test campaign included the following separate procedures:

- Barely visible impact damage (BVID) creation;
- Static loading up to LL;
- Fatigue loading;
- Static loading up to LL;
- Static loading up to UL followed by continuous loading to failure.

Static loading up to LL and UL were performed using load steps (the first two steps were 30 kN, each next step was 20 kN). Ten seconds at each load level was selected as the equilibrium holding time during the static measurements. In case of static load increases from UL to failure, the loading force was continuously increased with a load rate of 1.5 mm/min.

Damage with a depth of 1 mm before relaxation was considered as BVID, based on the thermoset requirements; this should be less for thermoplastics, but not less than 0.25 mm before relaxation or 0.3 mm after relaxation. The dent depth and the geometry were measured using a detailed visual inspection, UT A-scans, and dial depth gauge.

Static and fatigue loading were defined as follows:

- Static limit load (LL) was represented by an overpressure value of 1.3 DP (921 mbar). This value corresponded to a load force of 115 kN.
- Static ultimate load (UL) was represented by an overpressure of 2 DP (1386 mbar).
- Maximum force during fatigue loading (DPFAT) corresponded to an overpressure value of 1.17 DP (853 mbar). Fatigue harmonic loading with constant amplitude and sinusoidal force cycle were applied for up to 180,000 cycles. The maximum force in the load cycle DPFAT was 101 kN with a stress ratio of 0.1 (minimum/maximum load level). The fatigue load was applied with a test frequency of 0.75.

Loading forces include knock-down factors defined in airworthiness regulations.

Visuals and ultrasonic NDI inspections were done before and after each of the test phases and at pre-selected intervals during fatigue loading.

All the procedures were conducted under laboratory conditions (23 ± 5 °C).

## 5. Experiment

The experimental structure verification procedure, considering the damage tolerance philosophy, consisted of following parts:

- Impact damage test;
- Static and fatigue tests;
- Non-destructive testing.

Low velocity impact tests were conducted using the mass-drop method. To this end, a VZLU SUPR drop tower was used. Impact tests were performed using a semicircular indenter with a diameter of 25.4 mm and impact energy up to 45 J. 

**Figure 8:**
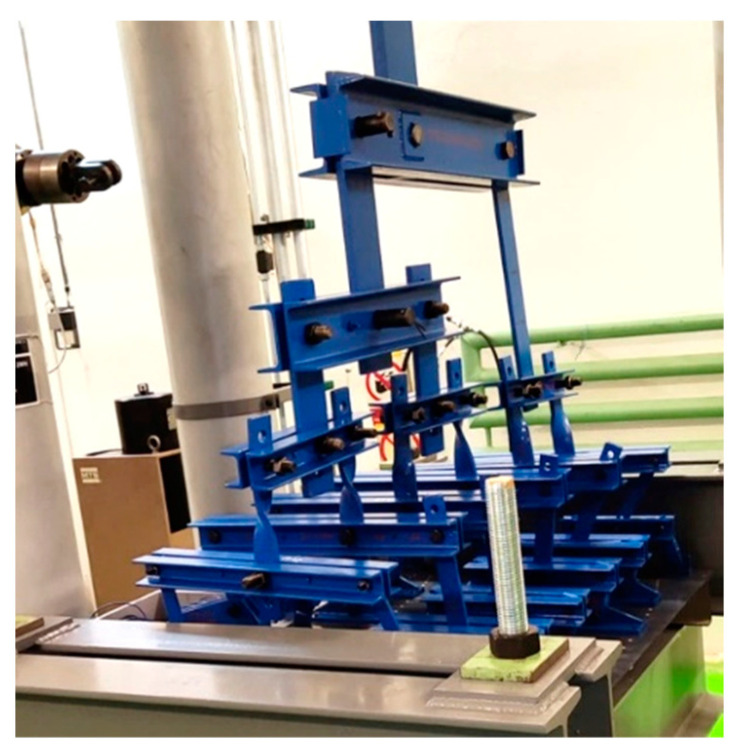
Whiffle tree loading system.

Static and fatigue testing was conducted using a uniaxial/biaxial VZLU four column test facility with a 2 MN maximum tensile force capacity. An INOVA EU3000 two channel electronic control system for real time measurement and control was used as an easy-to-use servo-controller for general testing applications. The frame, whiffle tree loading system, and panel configuration are shown in Figure 8.

During the experimental work, the following systems were used for data acquisition and measurements:

- Deformation optical measurement system;
- Strain gage measurement system;
- LVDT measurement system.

**Figure 9:**
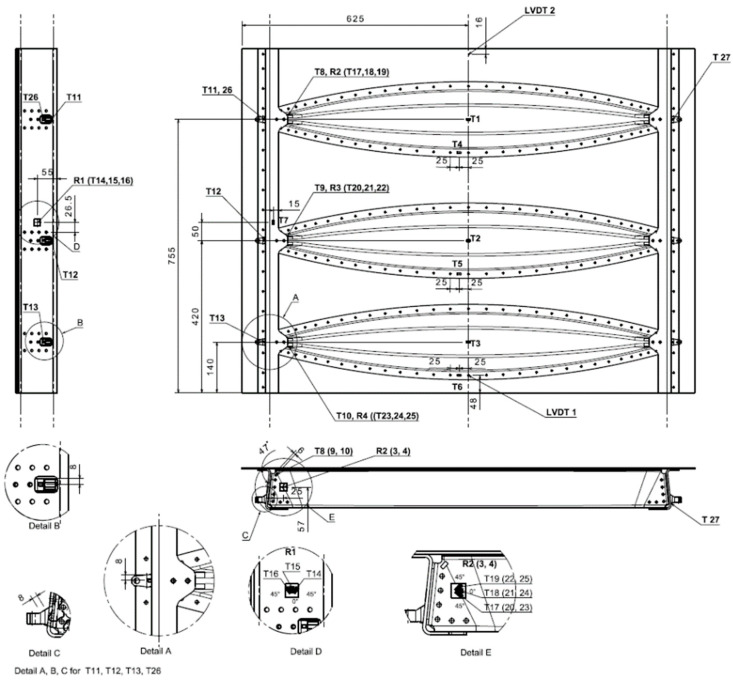
Strain gauges and LVDT network.

**Figure 10:**
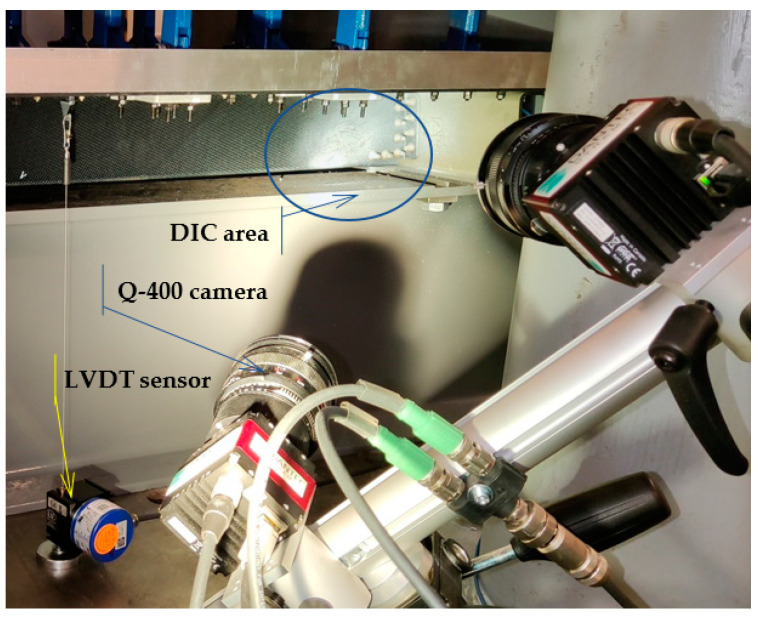
Arrangement of Q-400 measurement system and LVDT sensor no. 2.

A digital image correlation (DIC) DANTEC Q-400, BMCM measurement system for strain measurements and a MICRO EPSILON wire sensor system, WDS-1000-P60-CR-TTL, were used for data acquisition and evaluation. In sum, 15 uniaxial strain gauges, 4 rosettes (27 measured channels), and two LVDT sensors were installed. A scheme of the location and numbering of the individual sensors is illustrated in Figure 9. The elliptical blue pattern in Figure 10 labels the measured DIC critical area (Omega profile in connection with stop fitting).

## 6. Results

### 6.1. BVID

**Figure 11:**
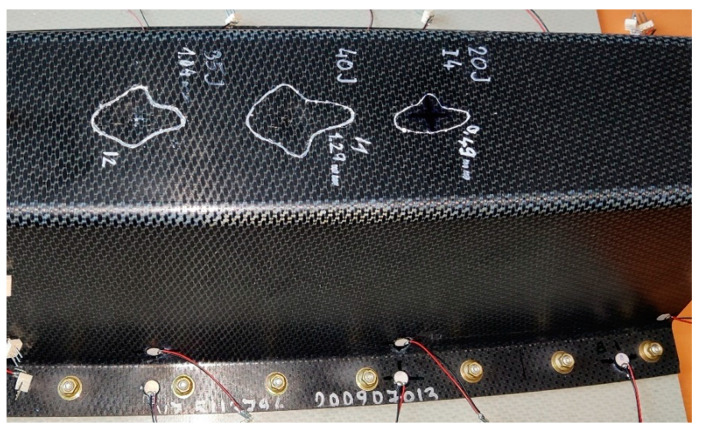
Small panel with damage from three impacts.

**Figure 12:**
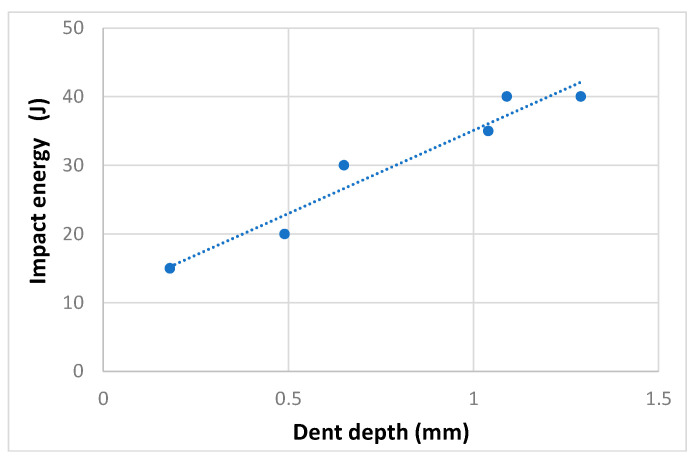
Impact energy vs. dent depth dependence of small panels.

First, impact energy calibration tests were done using two small panels, representing the skin/Omega beam joint. Three impact tests were conducted per small panel. Damaged areas were defined using an ultrasonic method (A-scan) and visualized using permanent marking. Figure 11 shows the impact damage in the first calibration panel. Table 2 documents the dent depth vs. impact energy dependence, measured after the impact tests; Figure 12 illustrates these measurements in a graphical form. Calibration tests resulted in 35 J impact energy for a dent depth of 1 mm.

**Figure 13:**
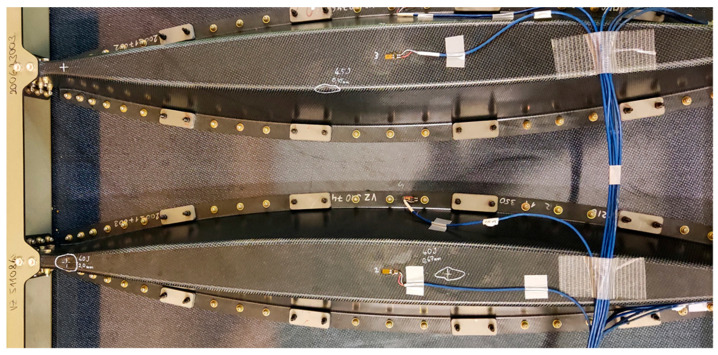
Overview of impact damage in the door panel.

Considering a slightly different door panel stiffness compared with the small panel arrangement and previous skills, 40 J of energy was chosen as a base for door panel impact. Three impact points were created in different areas of the Omega profiles of the door panel (close to stop fitting, in the middle of the Omega flange, and in the radius). A general view of two omega profiles of a door panel with impact damage is shown in Figure 13. Due to various local panel stiffnesses, the dent depths of the impact damage differed. The impact damage test in the radius of the Omega profile web was repeated with a higher impact energy (45 J); therefore, the dent depth of the first impact with an energy of 40 J was significantly smaller than expected (0.35 mm). An overview of the dent depth of individual impact damage points is documented in Table 3.

### 6.2. Mechanical Loading

**Figure 14:**
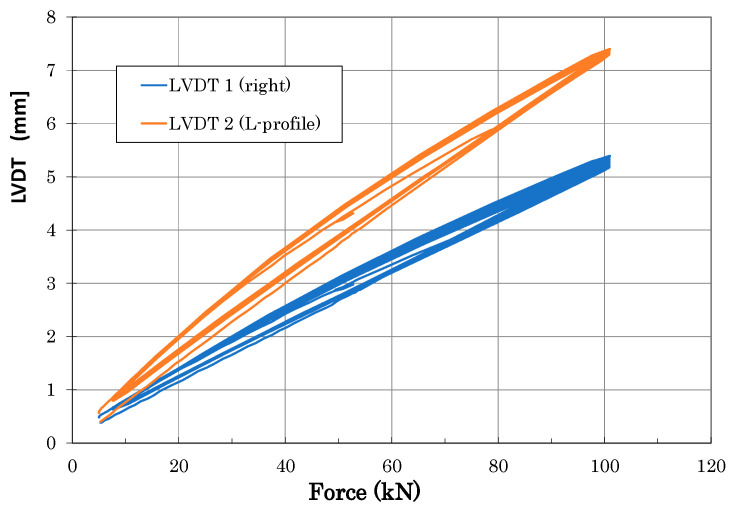
Example of typical LVDT curves measured during fatigue tests (measurement points are defined in Figure 9).

**Figure 15:**
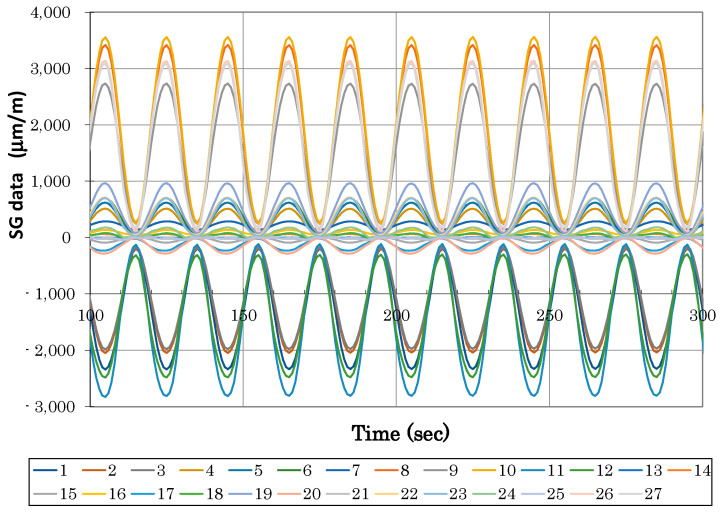
Typical strain gauge curves measured during fatigue test (x in legend corresponds to strain gauge Tx in Figure 9).

Strain and LVDT data were recorded during static loading at predefined load steps and continuously during fatigue cycling. Figure 14 and Figure 15 illustrate an example of typical LVDT (deformation vs. force dependence) and SGs (strains vs. time dependence) data recorded during fatigue loading. Individual measurement points of LVDT and SGs correspond to labelling defined in Figure 9. The various strain gauge curves in Figure 15 correspond to various locations and stress conditions (tension, compression) in various areas and parts of the door panel.

**Figure 16:**
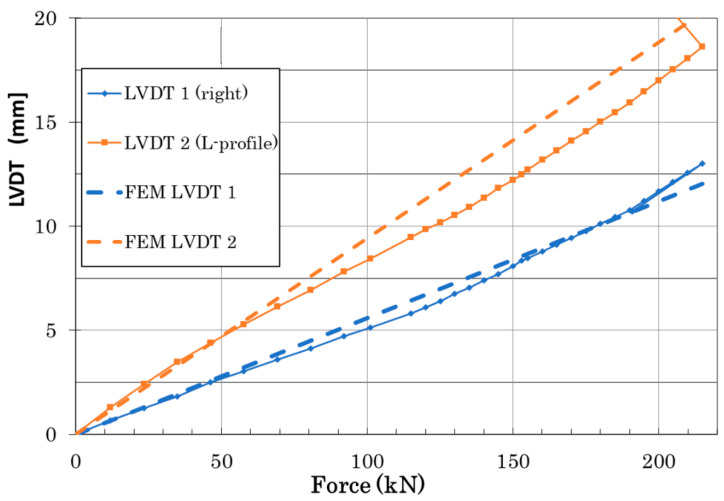
Force vs. LVDT displacement comparison of experimental data and numerical predictions.

**Figure 17:**
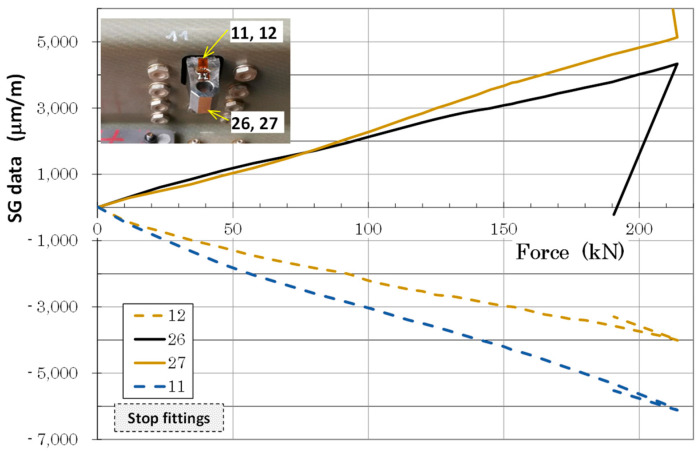
SG data and stop fittings up to door panel failure.

Load vs. deformation dependence up to door panel failure was measured using LVDT sensors placed in the middle of the panel span, as shown in Figure 16 (full lines). Strain measurements in the highly loaded part of the panel (surrounding of fittings) up to failure using resistance strain gauges are graphically displayed in Figure 16. Panel failure occurred at 214.01 kN in an area of the stop fitting connection with the Omega and Z-profiles (area of stop fitting with installed SGs corresponded to strain dependence in Figure 17).

The door panel sustained all required procedures and loading without structural failure. No damage propagation was observed. Real structural strength was about 25% higher compared with the ultimate (UL) structure strength.

## 7. Discussion

**Figure 18:**
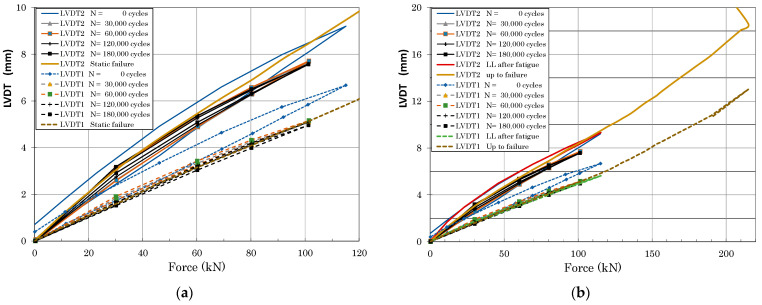
Comparison of panel deformation measured using LVDT sensors at various stages of the fatigue test ((**a**) loading up to DPFAT and LL, (**b**) complete data including loading up to panel failure).

**Figure 19:**
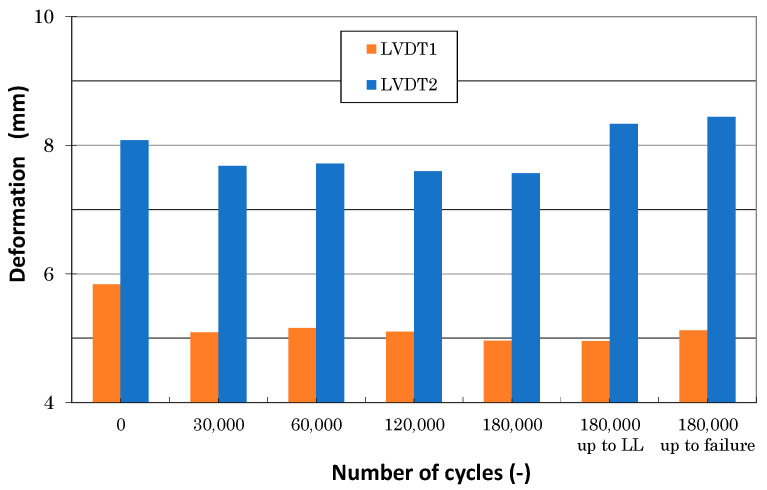
Comparison of panel displacement measured at DPFAT load in various stages of the testing campaign.

**Figure 20:**
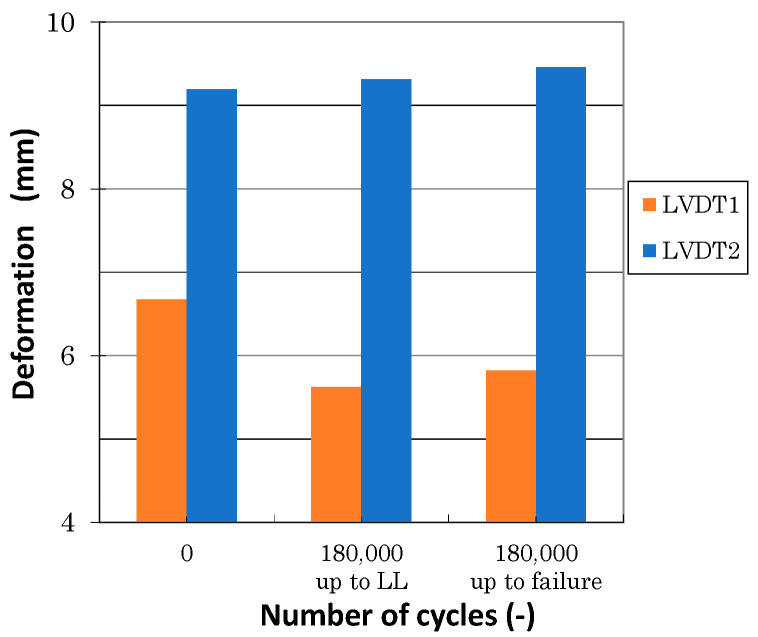
Comparison of LVDT1 and LVDT2 displacement measured at LL before and after fatigue testing.

Figure 18 illustrates the load-deformation curves measured using LVDT sensors during various stages of the experimental work. LVDT1 data showed lower values compared with LVDT2 data. It followed expectations due to nonlinear load distributions in various cross sections. Additionally, LVDT1 values measured before, during, and after fatigue loading differed. Displacement measured during and after fatigue was about 18% lower compared with data measured before fatigue (see Figure 19). This can be attributed to clearance adjustments. No significant differences in LVDT1 data measured during and after fatigue were observed (±1%). Similarly, LVDT2 values measured before fatigue loading differed slightly from the data measured during the fatigue stage; the difference was about 6%. A very important observation was the difference between displacement measurement immediately after fatigue and repeated measurements after NDI inspections (after approximately 16 days). Repeated measurements at time intervals showed higher values and absolute values that were very similar to the values measured before the start of fatigue loading. This conclusion can be also drawn from Figure 20. This behavior leads to nonlinear behavior and a relaxation in thermoplastics. Stiffness degradation of composites was studied in [34,35,36,37]; nevertheless, this needs additional detailed analyses.

**Figure 21:**
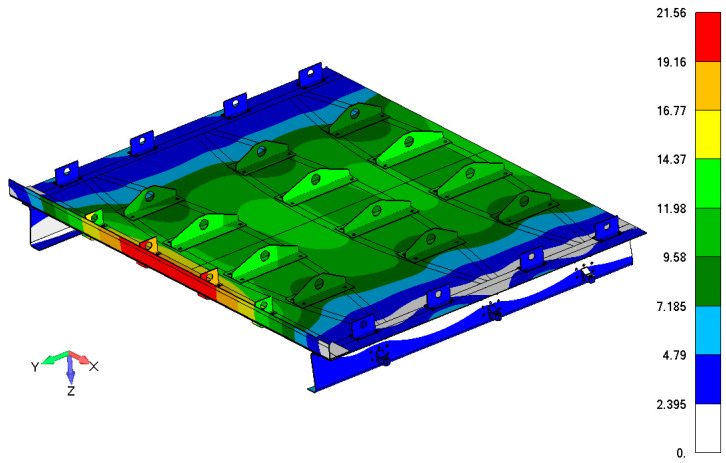
Contour map of total displacement (mm) corresponding to load level of 215 kN.

**Figure 22:**
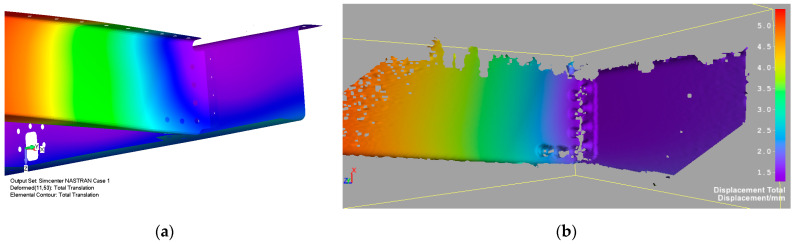
Comparison of total displacement contour map calculated using FE model (**a**) and measured using DIC system (**b**) before beginning of fatigue loading for LL level.

**Figure 23:**
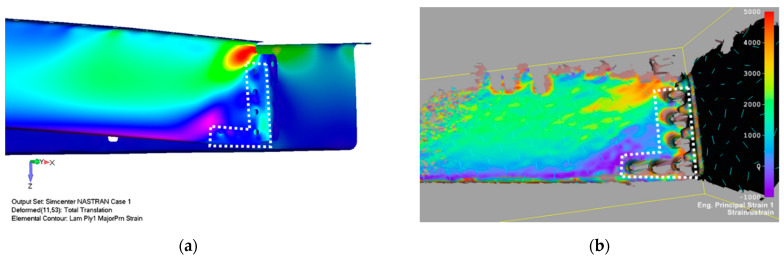
Comparison of principal strain contour map between FE simulation (**a**) and DIC measurement (**b**) before fatigue load for LL level. The dotted white line illustrates the area corresponding to the low resolution of the DIC measurement due to sharp lines or curved surfaces.

Numerically calculated displacement of the FE door panel model, using linear analysis corresponding to a failure load, is shown in Figure 21. Figure 16 illustrates a comparison of experimental data and the numerically predicted displacement at LVDT points. LVDT1 data and numerical prediction show good agreement. The displacement difference between numerical and experimental data in the LVDT2 point (reinforced area) was about 16%. A probable reason for this difference could be seen in the nonlinear behavior of real aluminium stiffeners due to their small plastic deformation, occurring above a load level of 50 kN. An additional cause of this difference could be load redistribution in the panel structure due to clearances and stiffness changes discussed above. Figure 22 and Figure 23 show comparisons of the total displacement and principal strain behavior calculated using the FE model and measured using a contactless optical system before the beginning of fatigue loading. Calculated and measured displacement and strain distributions in the selected critical area are depicted in Figure 22 and Figure 23 and are in good correlation.

**Figure 24:**
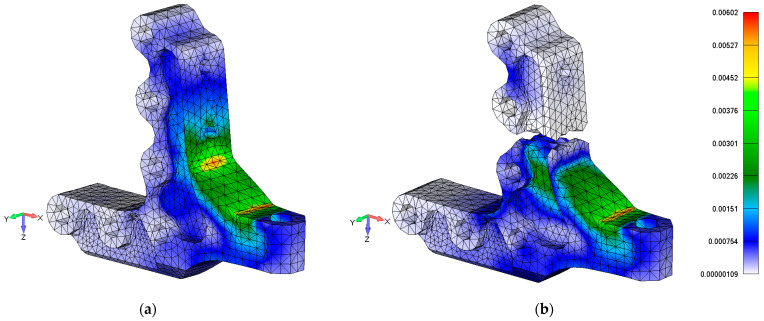
Comparison of principal strain contour map between FE simulation of the pristine stop fitting (**a**) and damaged stop fitting (**b**).

Figure 24 shows a comparison of the post-test simulation study of fatigue damage of stop fitting influences on strain behavior. The left side of the model in Figure 24 illustrates the strain distribution of a pristine stop fitting (a) and the model on the right side illustrates the strain distribution of the failed stop fitting (b).

A comparison of the pristine and damaged hinge models shows a decrease in the strain in the strain gauge area of about 6.5%. The probable reason for this difference is the simplification of fastener modelling and the application of a non-dense mesh and linear tetra-type element from the point of view of a global FE model approach.

**Figure 25:**
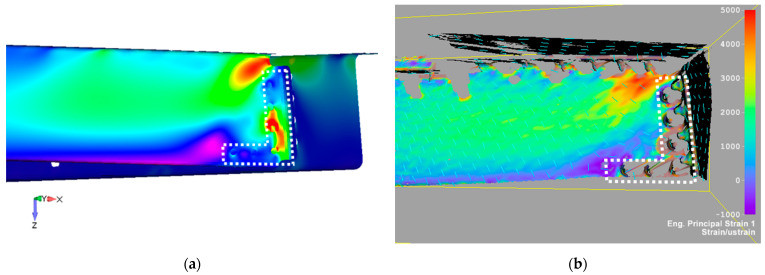
Comparison of principal strain contour map between the FE simulation (**a**) and DIC measurement (**b**) after a fatigue load for the LL level (115 kN) considering fatigue damage of the hinges (crack no. 1).

Figure 25 shows a study of the influences of post-test simulation of a damaged stop fitting on the strain behavior of a composite beam in the critical area. Composite beam strain distribution considering damage was similar compared with the strain distribution without damage measured before fatigue loading (see Figure 23). The probable reason for the small differences was good load redistribution in the composite material and the impossibility of achieving detailed strain distribution measurement using an optical system close to the holes for fasteners (washers, nuts).

Generally, the FE model shows a good correlation with the experimental verification. For improvements in the correlation between the test and simulation it is recommended to use a detailed model of fasteners, a nonlinear material model of metal parts, and nonlinear analysis (large displacement and rotation).

Future work should focus on the inspection of critical part improvements and pursue weight and costs saving. Possible procedures could be additional higher utilization of material mechanical properties (through decreased knock-down factors for example) and application of advanced damage tolerance philosophy through bonding or welding, together with a decrease in mechanical joints [38,39].

## 8. Conclusions

A new thermoplastic carbon composite panel door design, its manufacture, numerical analyses, and experimental verification according to the damage tolerance philosophy is presented. Thermoforming technology was used for the composite frame with variable cross section manufacturing. The variable shape of the Omega profile was optimized and manufactured without any imperfections (typically the occurrence of wrinkles in the thermoforming process is very dangerous). 

No impact damage propagation was observed in the composite parts during mechanical tests (180,000 fatigue cycles under a load level close to the limit load) despite visible impact damage being introduced into the structure. No significant difference between numerical simulations and the experimentally measured total deformation was observed.

Repeated deformation measurements over a time interval during fatigue showed changes and a nonlinear structure behavior. This can be attributed to the relaxation of the thermoplastics. The stiffness degradation of the thermoplastic composites needs future additional detailed analysis.

As there was an expectation that no growth of impact damage would be observed, this leads to the conclusion that the composite structure was overdesigned. Accordingly, in general, future work should be focused on improving the design philosophy to achieve weight and costs saving. Two ways to achieve this seem to be suitable: higher utilization of material mechanical properties (decrease of knock-down factors) and improving damage tolerance design (change the non-growth design criterion using the slow growth criterion, for example).

## Figures and Tables

**Table 1:** Overview of composite materials used for door panel manufacturing.

Structure Element	Material	Resin System	Nominal Thickness (mm)	Number of Layers	Layup
Omega profile	TC1100	PPS	3.1	10	[[(0,90)/(±45)]_2_/(0,90)]_s_
Z-profile	TC1100	PPS	4.34	14	[[(0,90)/(±45)]_3_/(0/90)]_s_
Skin	TC1100	PPS	3.41	11	[(0,90)/(±45)]_5_/(0,90)

**Table 2:** Overview of impact damage in the Omega profile.

Small Panel ID	Impact Energy (J)	Dent Depth (mm)
1	20	0.49
	40	1.29
	35	1.04
2	40	1.09
	15	0.18
	30	0.65

**Table 3:** Overview of impact damage in the Omega profile.

Impact Damage Location	Impact Energy (J)	Dent Depth (mm)
Middle of inner flange	40	0.67
Near to stop fitting	40	2.00
Radius	45	0.75

## Data Availability

Not applicable.
